# Innate immune responses at the asymptomatic stage of influenza A viral infections of *Streptococcus pneumoniae* colonized and non-colonized mice

**DOI:** 10.1038/s41598-021-00211-y

**Published:** 2021-10-18

**Authors:** Fabian Cuypers, Alexander Schäfer, Sebastian B. Skorka, Surabhi Surabhi, Lea A. Tölken, Antje D. Paulikat, Thomas P. Kohler, Saskia A. Otto, Thomas C. Mettenleiter, Sven Hammerschmidt, Ulrike Blohm, Nikolai Siemens

**Affiliations:** 1 Department of Molecular Genetics and Infection Biology, University of Greifswald, Greifswald, Germany; 2 Institute of Immunology, Friedrich-Loeffler-Institut, Federal Research Institute for Animal Health, Greifswald - Island of Riems, Germany; 3 Institute for Marine Ecosystem and Fisheries Science (IMF), Center for Earth System Research and Sustainability (CEN), University of Hamburg, Hamburg, Germany

**Keywords:** Immunology, Infection, Infectious diseases, Innate immune cells, Innate immunity, Bacteria, Bacterial host response, Microbiology, Virology, Influenza virus

## Abstract

Seasonal Influenza A virus (IAV) infections can promote dissemination of upper respiratory tract commensals such as *Streptococcus pneumoniae* to the lower respiratory tract resulting in severe life-threatening pneumonia. Here, we aimed to compare innate immune responses in the lungs of healthy colonized and non-colonized mice after IAV challenge at the initial asymptomatic stage of infection. Responses during a severe bacterial pneumonia were profiled for comparison. Cytokine and innate immune cell imprints of the lungs were analyzed. Irrespective of the colonization status, mild H1N1 IAV infection was characterized by a bi-phasic disease progression resulting in full recovery of the animals. Already at the asymptomatic stage of viral infection, the pro-inflammatory cytokine response was as high as in pneumococcal pneumonia. Flow cytometry analyses revealed an early influx of inflammatory monocytes into the lungs. Neutrophil influx was mostly limited to bacterial infections. The majority of cells, except monocytes, displayed an activated phenotype characterized by elevated CCR2 and MHCII expression. In conclusion, we show that IAV challenge of colonized healthy mice does not automatically result in severe co-infection. However, a general local inflammatory response was noted at the asymptomatic stage of infection irrespective of the infection type.

## Introduction

Community-acquired pneumonia (CAP) is one of a most common lung infections acquired outside of the hospital. The annual incidence ranges from five to eleven cases per 1000 in human populations in Europe and North America^1^. *Streptococcus pneumoniae* (pneumococcus) remains the most commonly identified cause of CAP^2^. During seasonal influenza outbreaks, the circulating influenza A virus (IAV) becomes the major cause of CAP^3^. Several studies showed that IAV infections predispose the host to bacterial dissemination from the upper to the lower respiratory tract (URT, LRT)^4–6^. However, most clinical investigations linked severe co-infections to individuals with comorbidities, elderly people, pregnant women, and children under the age of one. In contrast, the majority of healthy individuals recover from such severe infections^7^.

The innate immune system is of crucial importance in limiting viral spread as well as combatting the onset of subsequent bacterial infection of the lungs^3^. At early stages of infection, resident professional phagocytes and recruited neutrophils and monocytes are the first responders. Alveolar (AMs) and interstitial macrophages (IMs) reside within the lungs^8^. AMs are mainly involved in pathogen sensing and rapid recruitment of leukocytes^9^. IMs are less abundant and exhibit reduced phagocytic potential. Several studies have shown that IAV infection leads to a depletion of AMs, resulting in pneumococcal overgrowth, severe tissue pathology, and fatal outcome^10,11^. However, it was also demonstrated that interleukin (IL)-1 signaling prevents AM depletion and therefore, AMs are critically involved in host resistance to co-infections^12^. In contrast, the role of IMs is not clear yet. Dendritic cells [DCs; conventional DCs (cDCs) and plasmacytoid DCs (pDCs)] respond rapidly to an infection^13^ by producing type I interferons (IFNs)^14,15^. Only a few studies analyzed the role of DCs in IAV and pneumococcal co-infections. While one study showed that IAV infection results in reduced cDC counts in lungs and, consequently, predisposes mice to a severe co-infection^16^, another study demonstrated elevated levels of lung DCs post IAV challenge and a potentially protective effect^17^.

Neutrophils and monocytes are also rapidly recruited to the site of infection^18,19^. In the hyper-inflammatory environment, recruited monocytes can differentiate to DCs/macrophages or directly phagocytose pathogens. A sustained increase of pro-inflammatory monocytes was found in IAV-infected patients^20^. A pivotal role of both recruited cell types in pneumococcal and IAV co-infections was suggested. Pneumococcal carriage was associated with a quick influx of neutrophils and a subsequent recruitment of monocytes, while challenge of colonized individuals with live-attenuated IAV impaired these immune responses^21^.

The murine studies provided valuable insights into innate immune responses during bacterial and viral co-infections. However, they also have limitations. In most cases the chosen order of infection, first IAV followed by pneumococci, might not reflect a natural sequence of co-infections. Furthermore, most studies focus on one immune cell type during a severe phase of infection. Here, we characterized the innate immune cell imprint of the lungs in response to natural co-infections. We show that pneumococci asymptomatically persist in the nasopharyngeal cavity of mice. IAV infection of colonized mice results in trafficking of the bacteria to the lungs in 50% of cases. However, the animals recover after a bi-phasic course of disease. The early asymptomatic state of infections was characterized by elevated cytokine levels irrespective of the infection type. Analyses of the innate immune cell composition showed infection-driven imprint with no differences between colonized and non-colonized mice after viral challenge. However, already at the asymptomatic stage, the inflammatory response was as high as in severe bacterial pneumonia.

## Results

### Intracellular killing of pneumococci by professional phagocytes is not affected by IAV infection

In the lungs, resident and recruited immune cells are first challenged with IAV prior to subsequent bacterial exposure. To mimic this order of infection, professional phagocytes were challenged with pathogens in this particular order. To investigate if IAV infection affects the ability of professional phagocytes to kill pneumococci, susceptibility of human primary monocytes and macrophages to H1N1 was analyzed (Fig. S1A). Based on these results, a MOI of 0.1 was used for subsequent co-infection experiments. All three cell types were infected with colonizing pneumococcal strain 19F. Irrespective of prior H1N1 infection, intracellular bacteria were eliminated by phagocytes. No differences in bacterial killing between mono- and co-infected cells were observed (Fig. S1B–D). In addition, pathogen eliminating properties of mouse monocytes/macrophages were tested. First, IAV replication was confirmed in J774 cells (Fig. S1E). Analyses of pneumococcal killing in single and co-infected J774 cells mirrored the human phenotype. However, a 19 h delay in bacterial elimination was noted (Fig. S1F). These results suggested that IAV infection does not impair the general killing function of these cell types.

### Streptococcus pneumoniae 19F colonizes C57BL/6J mice

**Figure 1:**
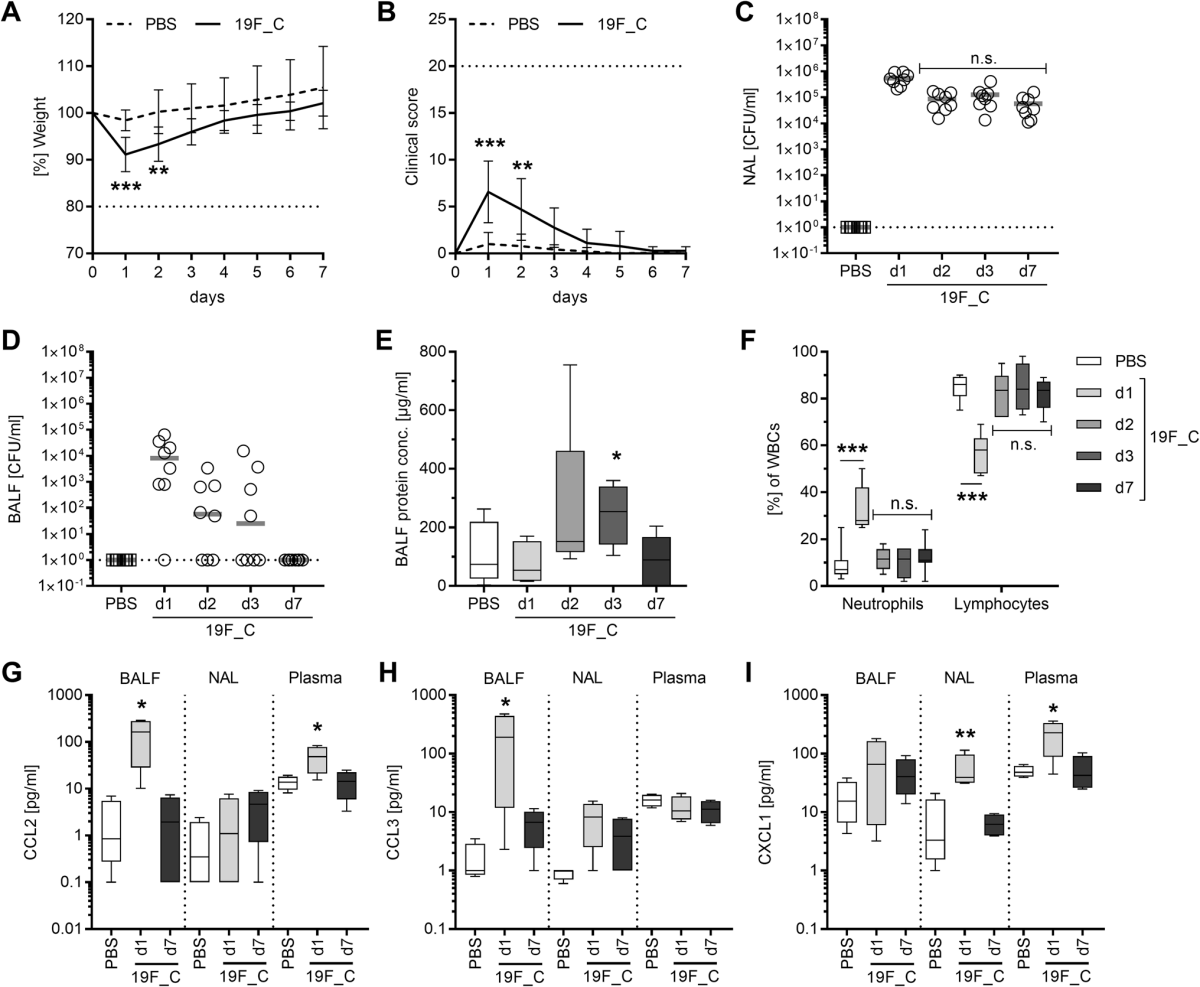
C57BL/6J mice are susceptible to *S. pneumoniae* 19F colonization. Female C57BL/6J mice were intranasally colonized with 1 × 10^7^ CFU of *S.* *pneumoniae* 19F (19F_C) or challenged with PBS and monitored over seven consecutive days. (**A**) Weight and (**B**) clinical score were monitored daily. Horizontal dotted lines in A and B indicate termination conditions (20% weight loss and/or clinical score of 20) of the experiment. CFU counts in (**C**) nasal washes (NAL) and (**D**) bronchoalveolar fluid (BALF), (**E**) protein concentration in the BALF, (**F**) the white blood cell differential (WBC) limited to neutrophil and lymphocyte counts as well as (**G**–**I**) chemokine release were determined at indicated time points. Horizontal dotted lines in (**C**) and (**D**) indicate the limit of detection. Two independent experiments with four mice per group (total: n = 8) were performed. In general, mean values ± SD are displayed (**A**, **B**). Each dot represents one mouse and horizontal lines display mean values (**C**–**E**). The data in (**F**–**I**) are displayed as box plots. The level of significance between the PBS and all other groups was determined using Kruskal Wallis test with Dunn’s multiple comparison post-test (n.s., not significant, *p < 0.05; **p < 0.01; ***p < 0.001). Dotted lines in A and B reprsent.

To mimic natural co-infection, it was of crucial importance to use a pneumococcal strain, which is able to colonize mice. Invasive pneumococcal strains (e.g., TIGR4) either cause acute severe infections or are cleared if low initial inoculum is used^22^. Therefore, colonizing properties of *S.* *pneumoniae* 19F strain, a serotype, which is typically associated with human colonization, were assessed. Colonization was defined as stable bacterial load in the URT and absence of local and systemic inflammation. Initial colonization was characterized by a weight drop and a mild increase in clinical score on day one post bacterial application. Both parameters normalized over the next three days (Fig. 1A,B). *S.* *pneumoniae* 19F stably colonized mice for seven consecutive days with no detectable bacteria in the lungs on day seven (Fig. 1C,D). To exclude a sustained local or systemic inflammatory response, protein and chemokine concentrations as well as white blood cell differential (WBC) were determined (Fig. 1E–I). The initial increase in local and systemic inflammation normalized within three days post colonization.

### Non-mouse adapted H1N1 causes mild symptoms of viral pneumonia in C57BL/6J mice

**Figure 2:**
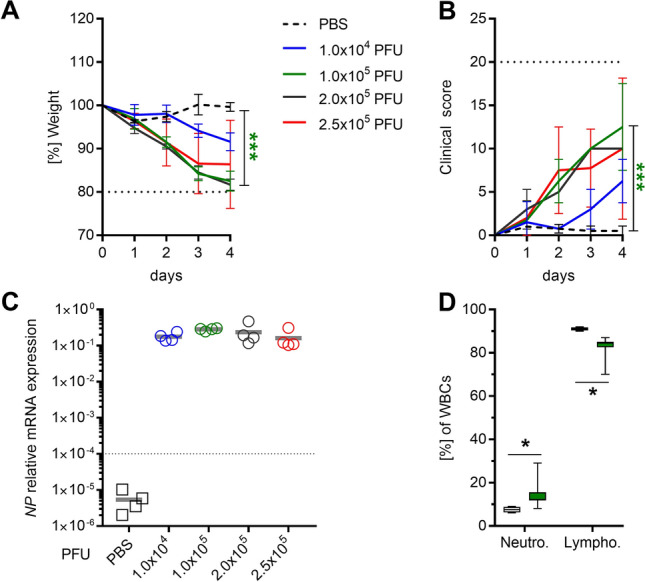
C57BL/6J mice are susceptible to non-mouse adapted H1N1 IAV. C57BL/6J were intranasally inoculated with indicated PFUs of H1N1 or challenged with PBS and monitored over 4 consecutive days. (**A**) Weight and (**B**) clinical score were determined daily. Horizontal dotted lines in A and B indicate termination conditions (20% weight loss and/or clinical score of 20) of the experiment. (**C**) On day 4, lungs were extracted and viral infection was confirmed via IAV *NP*-specific qRT-PCR (dashed line indicates detection limit). (**D**) WBC limited to neutrophil and lymphocyte counts were determined at day 4. One experiment with four mice per group (total: n = 4) was performed (**A**–**D**). In general, mean values ± SD are displayed (**A**, **B**). Each dot represents one mouse and horizontal lines display mean values (**C**). The data in (**D**) are displayed as box plots. The level of significance between the PBS and all other groups in (**A**) was determined using Kruskal Wallis test with Dunn’s multiple comparison post-test. In (**D**), Mann Whitney *U*-test was performed (*p < 0.05).

Mouse adapted IAV strains tend to cause severe pneumonia. To induce only mild symptoms, mice were infected with a non-mouse adapted H1N1^23^ and disease progression was monitored (Fig. 2). Dose-dependent weight loss and increase in clinical score was noted (Fig. 2A,B). On day four, the presence of viral RNA in lungs was confirmed (Fig. 2C). WBC showed elevated levels of neutrophils in circulation (Fig. 2D). Based on these results, 1 × 10^5^ PFU were used for all H1N1 infections.

### Local and systemic inflammatory responses at the early onset of pneumonia

**Figure 3:**
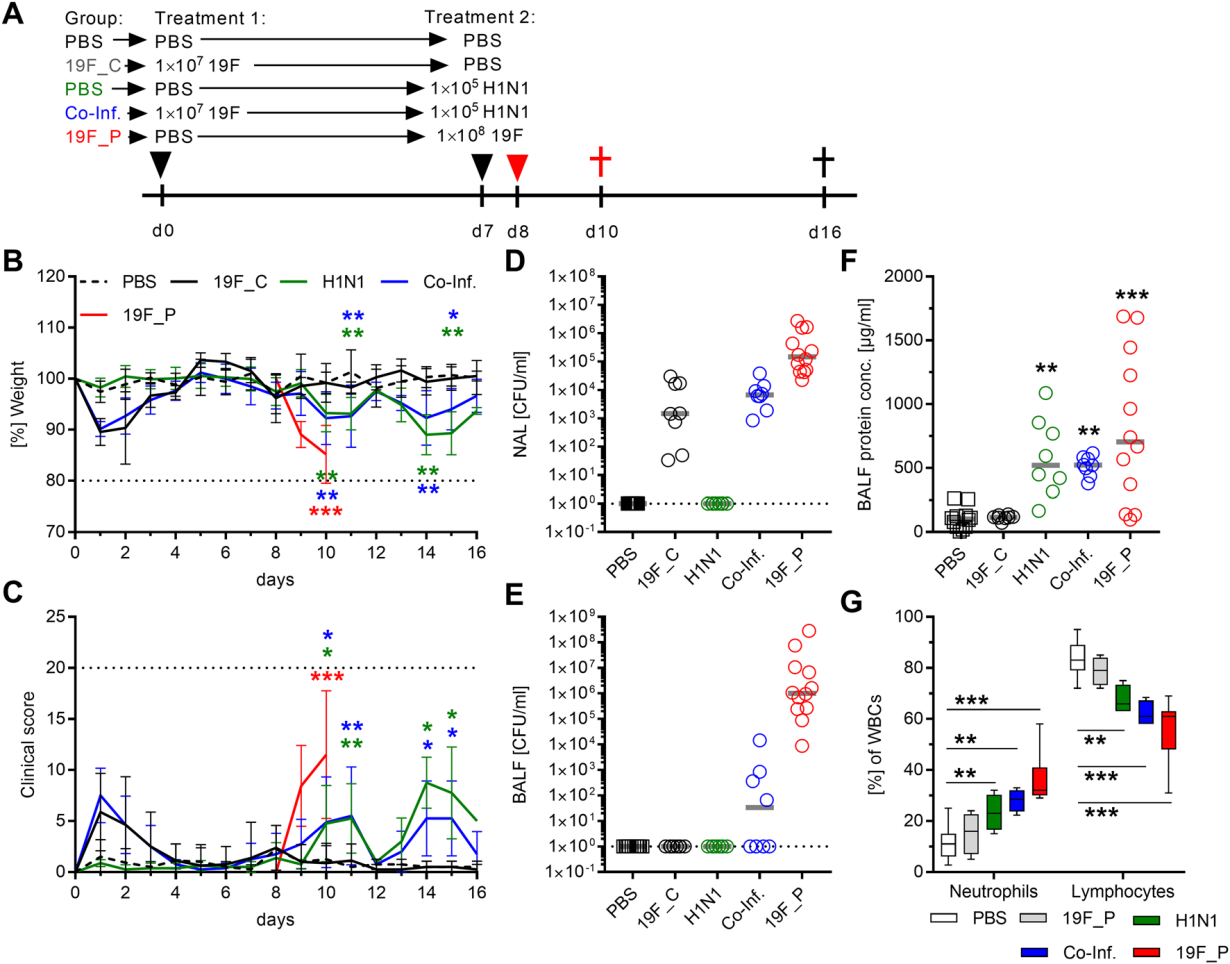
Mild H1N1 infection of *S.* *pneumoniae* 19F colonized mice does not result in severe co-infection. (**A**) Female C57BL/6J mice were intranasally colonized with *S.* *pneumoniae* 19F (1 × 10^7^ CFU) for seven days followed by a subsequent mild H1N1 infection (1 × 10^5^ PFU; Co-Inf.). PBS challenged (PBS), only colonized (19F_C), and only H1N1 infected (H1N1) mice served as controls. In addition, severe pneumococcal pneumonia was induced via intranasal inoculation of *S. pneumoniae* 19F (1 × 10^8^ CFU; 19F_P; day 8). (**B**) Weight and (**C**) clinical score were monitored over a period of 16 consecutive days. Horizontal dotted lines in B and C indicate termination conditions (20% weight loss and/or clinical score of 20) of the experiment. (**D**) CFU counts in nasal washes (NAL) and (**E**) in BALF, (**F**) protein concentration of the BALF and (**G**) WBC were determined after the animals were sacrificed. Horizontal dotted lines in (**D**) and (**E**) indicate the limit of detection. Animals were sacrificed at day 16 except for the pneumonia group which was sacrificed at day ten. Two independent experiments with four to six mice per group (total: n ≥ 8) were performed. In general, mean values ± SD are displayed (**B**, **C**). Each dot represents one mouse and horizontal lines display mean values (**D**–**F**). The data in (**G**) are displayed as box plots. The level of significance between the PBS and all other groups was determined using Kruskal Wallis test with Dunn’s multiple comparison post-test (*p < 0.05; **p < 0.01; ***p < 0.001).

Next, a mild H1N1 infection was induced seven days after bacterial colonization (referred to as co-infection). Single H1N1 infection served as a control. To track severe responses, a high dosage group of pneumococcus infection was included (Fig. 3A). In contrast to severe pneumococcal pneumonia, H1N1 infections of colonized and non-colonized mice were characterized by a bi-phasic course of disease resulting in full recovery (Fig. 3B,C). Analyses of bacterial burden in nasal washes (NAL) and bronchoalveolar fluid (BALF) showed that pneumococci remained restricted to the nasopharynx in colonized mice, whereas pneumococcal pneumonia was characterized by a high bacterial burden, and 50% of co-infected mice had bacteria in the lungs (Fig. 3D,E). Although local (Fig. 3F) and systemic (Fig. 3G) inflammatory responses were noted in all three infections, no significant differences between the groups were detected.

**Figure 4:**
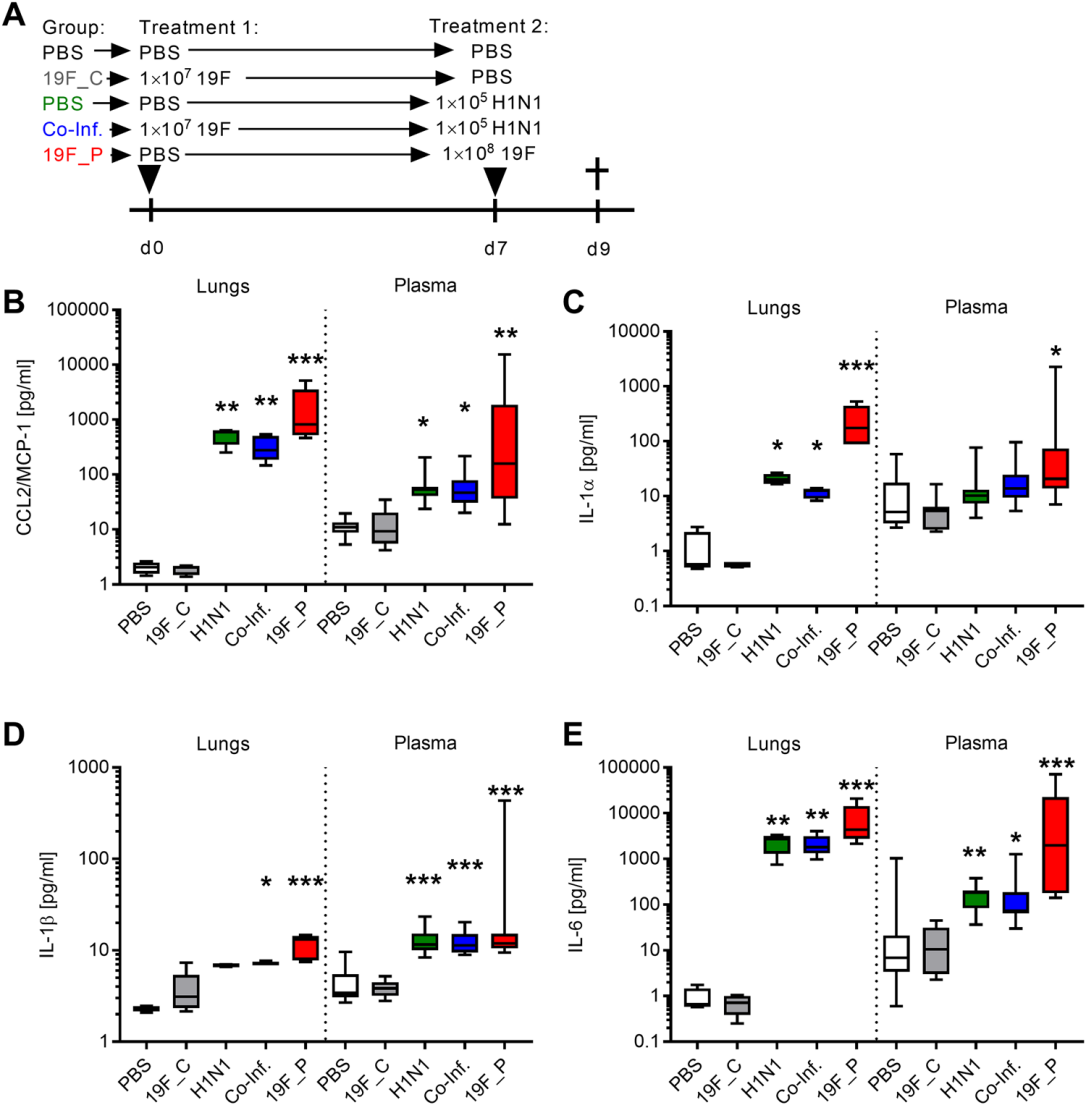
Local and systemic cytokine production in response to mono- and co-infections. (**A**) Scheme displaying the time line of different mice infections. Mice were sacrificed at day 9 and (**B**) CCL2/MCP-1, (**C**) IL-1α, (**D**) IL-1β, and (**E**) IL-6 levels were determined in lungs and plasma. Two independent experiments with six mice per group (total: n = 12) were performed. The data are displayed as box plots. The level of significance between the PBS and all other groups was determined using Kruskal Wallis test with Dunn’s multiple comparison post-test (*p < 0.05; **p < 0.01; ***p < 0.001).

Based on these results, we hypothesized that an early inflammatory response might rescue healthy mice from severe disease progression in viral and co-infections. All animals were sacrificed before the onset of clinical signs two days post viral infection (Fig. 4A and Supplementary Fig. S2). Although no visible signs of illness were noted in both H1N1-infected groups, 50% of co-infected mice had bacteria in the lungs and exhibited elevated systemic responses (Supplementary Fig. S2). Furthermore, elevated influx of leukocytes was noted in lungs of co-infected mice and particularly in bacterial pneumonia group (Supplementary Fig. S2). Next, cytokines were measured in lungs and plasma. Bacterial pneumonia resulted in elevated cytokine levels in both (Fig. 4). Only background levels of IL-10 and IL-12p70 were seen in all infected animals (Supplementary Fig. S3). Although both virus-infected groups were asymptomatic, almost equally high cytokine levels were detected locally and systemically as compared to single bacterial pneumonia, with the exception of IL-1α. Both viral infections had similar IL-1α levels. However, IL-1α was only elevated in the lungs (Fig. 4). In contrast, GM-CSF was detected in plasma of all infected mice and exclusively in lungs of the pneumococcal pneumonia group (Supplementary Fig. S3).

### Myeloid cell composition and their phenotype in the lungs in response to different infections

Although single virus and co-infected animals were clinically asymptomatic, an inflammatory cytokine response was noted in the lungs. This led us to explore the composition of myeloid cell subsets of the lungs. To distinguish between vascular and alveolar/interstitial lung compartments, mice received intravenous injection of labeled CD45 antibody. Consequently, CD45^hi^ cells were defined as cells from the vascular and CD45^lo^ as cells from the alveolar/interstitial compartment. Cell subsets were further classified as depicted in Supplementary Fig. S4.

**Figure 5:**
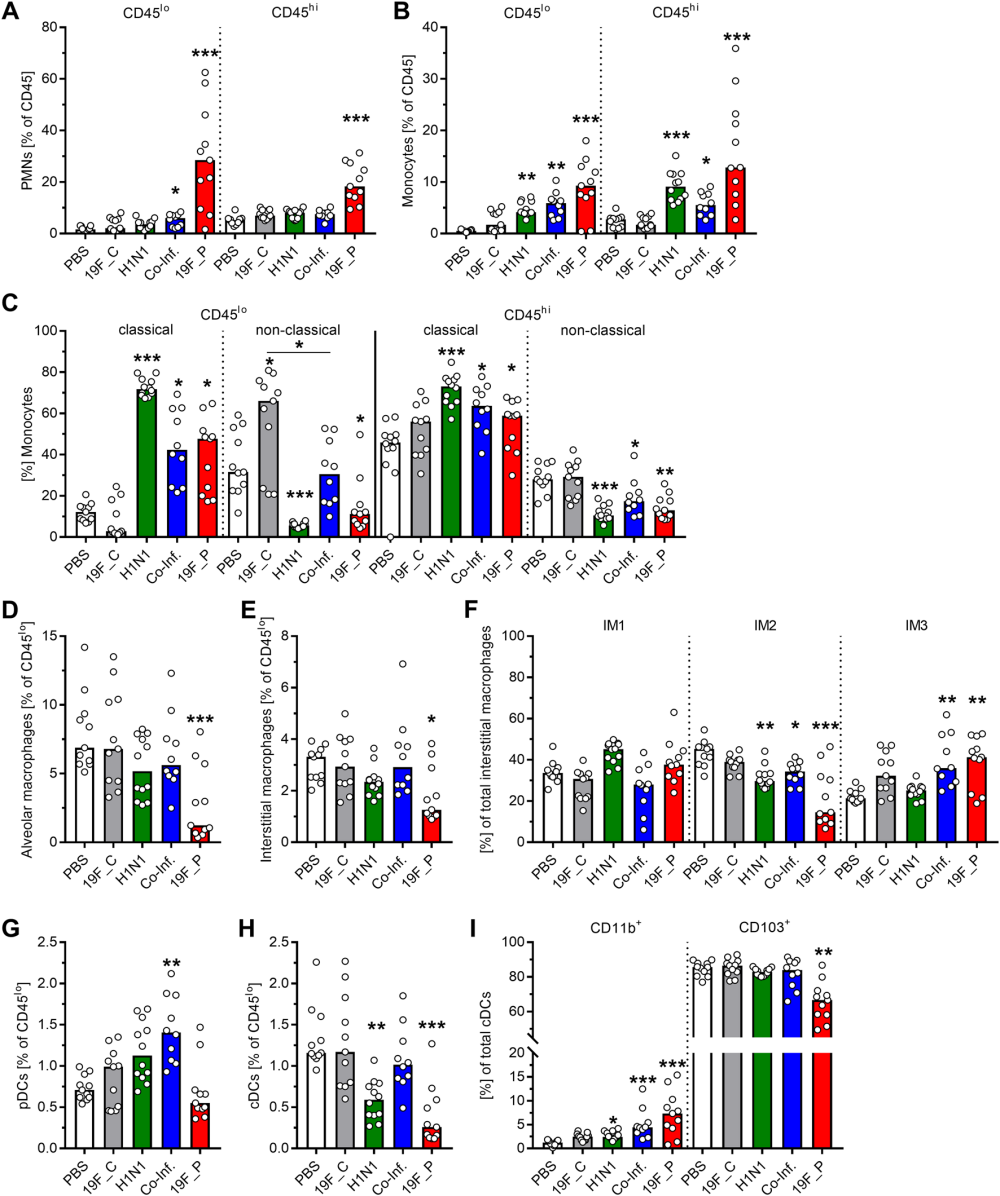
Innate immune cell composition of the lungs in response to mono- and co-infections. The infections were performed as displayed in (**A**). At day nine post infections, lungs were harvested, digested, and resulting single cell suspensions were analyzed via flow cytometry. CD45^lo^ cells were classified as cells from the alveolar/interstitial compartment and CD45^hi^ cells as cells from the vascular compartment of the lung. Frequencies of (**A**) neutrophils (PMNs), (**B**) total monocytes and (**C**) monocyte subsets, (**D**) alveolar macrophages (AMs), (**E**) interstitial macrophages (IMs) and (**F**) IM subsets, (**G**) plasmacytoid dendritic cells (pDCs), (**H**) conventional DCs (cDCs), and (**I**) cDC subsets are displayed. Two independent experiments with six mice per group (total: n ≥ 10) were performed. Each dot represents one mice and bars denote median values. The level of significance between the PBS and all other groups was determined using Kruskal Wallis test with Dunn’s multiple comparison post-test (*p < 0.05; **p < 0.01; ***p < 0.001).

In general, elevated frequencies of recruited neutrophils and monocytes were seen in both lung compartments of the bacterial pneumonia group (Fig. 5A,B). Neutrophil influx was slightly increased in co-infections (Fig. 5A). In contrast, increased numbers of monocytes were detected in lungs of both viral infections (Fig. 5B). Analyses of monocyte subpopulations showed that the total rise in monocytes could be linked to the influx of inflammatory/classical monocytes, while non-classical monocyte frequencies were reduced in all infections (Fig. 5C). Analyses of the resident macrophages showed that at the early onset of disease, viral infection did not lead to the depletion of AMs and IMs. In contrast, severe bacterial infection resulted in a drastic drop of both cell types (Fig. 5D,E). Based on the current models ^8^, three subsets of IMs were examined (Supplementary Fig. S4). Irrespective of the infection type, IM2 frequencies decreased. In contrast, IM3 frequencies increased in all bacterial infections (Fig. 5F). Furthermore, elevated frequencies of pDCs were exclusively detected in co-infected mice (Fig. 5G). In contrast, the total cDC population remained unaffected in co-infections. However, single viral and bacterial infections were characterized by reduced numbers of cDCs (Fig. 5H). Analyses of cDCs subpopulations revealed that the frequency of CD11b^+^ cDCs was increased in all infections, while CD103^+^ cDCs remained mostly unaffected (Fig. 5I).

**Figure 6:**
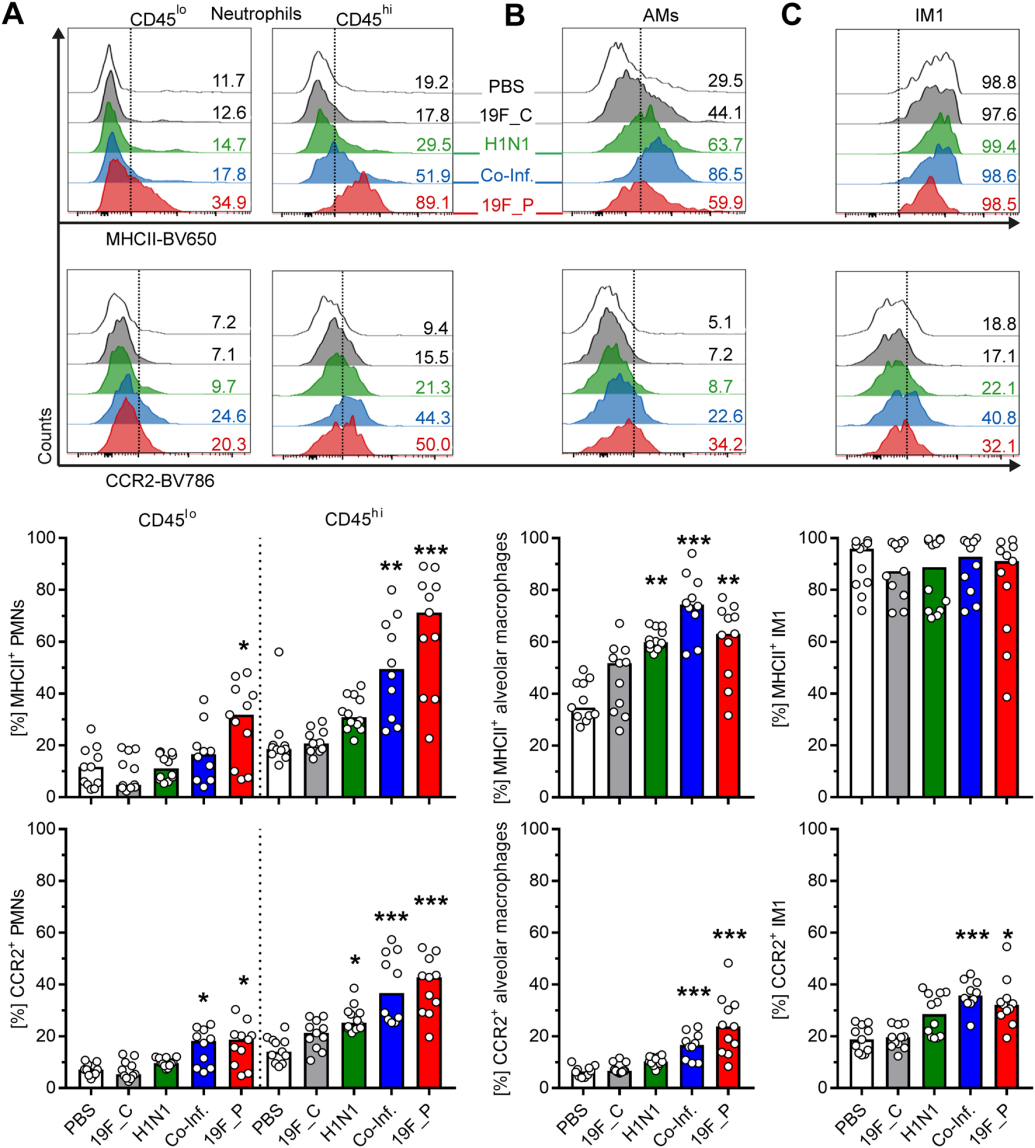
Expansion of MHCII^+^ and CCR2^+^ innate immune cells in response to bacterial and viral infections. The infections were performed as displayed in Fig. 5A. Representative histograms with numbers indicating marker positive cells (upper panel) and summary of MHCII (middle panel) and CCR2 (lower panel) expressing cells. The data summarizes responses of (**A**) neutrophils, (**B**) alveolar macrophages (AMs), and (**C**) type 1 interstitial macrophages (IM1). Each dot represents one mouse and bars denote median values (n ≥ 8). The level of significance between the PBS and all other groups was determined using Kruskal Wallis test with Dunn’s multiple comparison post-test (*p < 0.05; **p < 0.01; ***p < 0.001).

**Figure 7:**
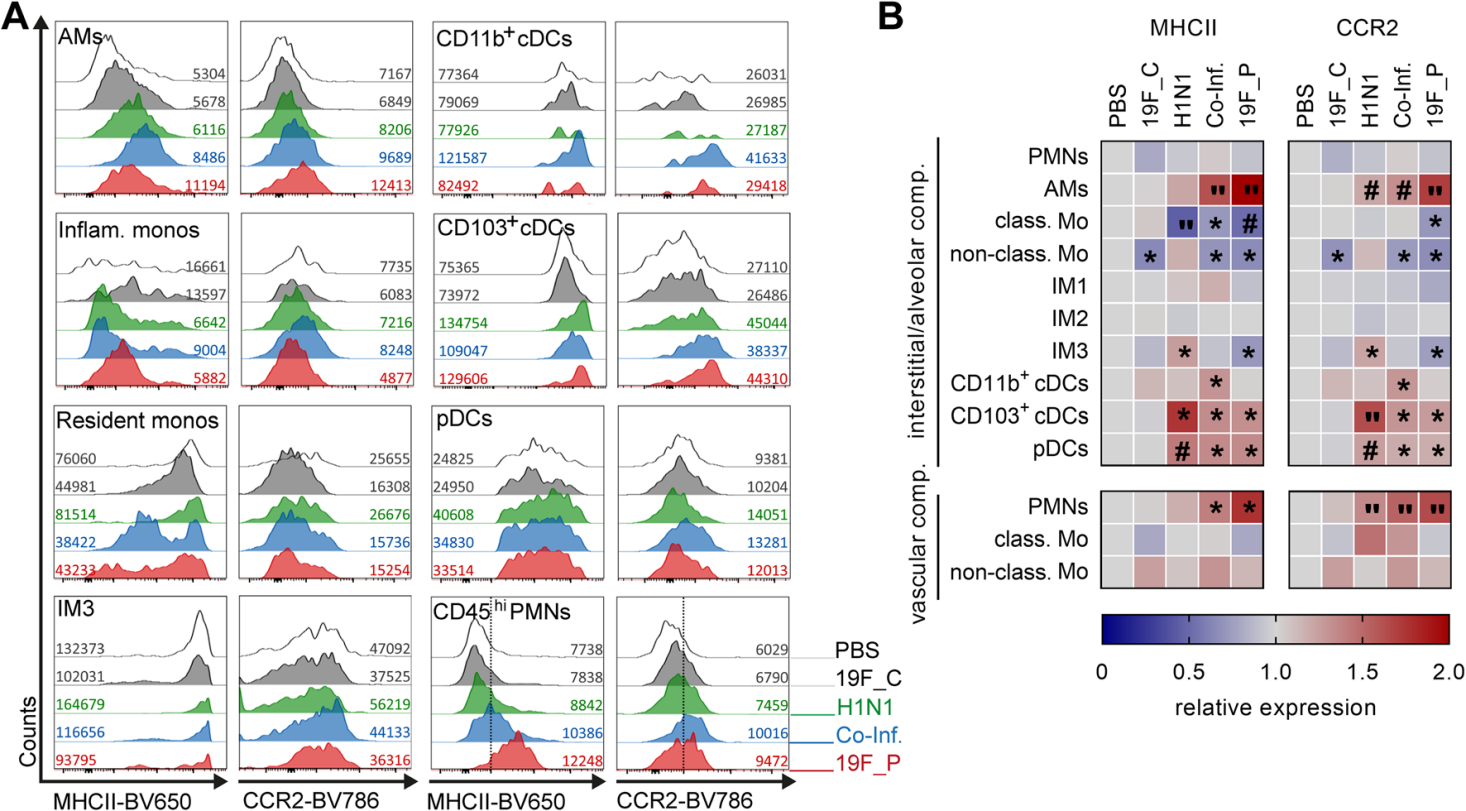
Innate immune cells display enhanced activation at early stages of pulmonary infections. (**A**) Representative histograms of flow cytometry analyses with numbers indicating mean fluorescence intensity (MFI) and (**B**) heat map analyses of relative MFI expression of indicated cell subsets in comparison to the PBS control (n ≥ 8). The level of significance between the PBS and all other groups was determined using Kruskal Wallis test with Dunn’s multiple comparison post-test (*p < 0.05; "*p* < 0.01; ^#^*p* < 0.001).

Recruitment of innate immune cells to the site of infection is mainly mediated by the detection of CCL2 via CC chemokine receptor 2 (CCR2)^24^. Furthermore, resident and recruited cells contribute to adaptive immune responses by presenting antigens via MHCII^25^. Therefore, these two markers of activation were analyzed. Elevated frequencies of MHCII and CCR2 expressing neutrophils, AMs, and CCR2^+^ IM1 were mostly detected in the lungs of single bacteria and co-infected mice (Fig. 6). However, no significant differences in frequencies of the innate immune cell subsets expressing MHCII and CCR2 were found in the majority of cell populations (Supplementary Fig. S5). To investigate the phenotype in more detail, the level of MHCII and CCR2 expression on all cells were analyzed (Fig. 7). Within the interstitial/alveolar compartment, MHCII and CCR2 levels were elevated on AMs, CD103^+^ cDCs, and pDCs in all infections. CD11b^+^ cDC showed an upregulation of both molecules exclusively in co-infections (Fig. 7). While in IM3 both proteins were upregulated in viral infection, the opposite regulation was noted in single bacterial infections. Furthermore, monocytes were characterized by lower levels of both molecules in response to the majority of infections (Fig. 7). Within the vascular compartment, only neutrophils showed elevated levels of MHCII and CCR2 in single bacterial and co-infections (Fig. 7).

## Discussion

Severe infections including CAP are characterized by an initial hyper-inflammation followed by immune paralysis^26^. It is assumed that IAV paves the way for colonizing bacteria to spread into the lower respiratory tract, resulting in high mortality^3^. However, most studies use initial viral application followed by bacterial infection. Since up-to one third of humans are asymptomatically colonized with *S. pneumoniae*^27^, our model potentially more closely resembles respiratory co-infections in humans. Our study shows that the onset of pneumonia in healthy mice correlates with a homogeneous immunological response. The onset of infection was characterized by elevated cytokine levels in lungs and plasma, and elevated influx of neutrophils and monocytes in lungs. The majority of immune cells displayed an activated phenotype.

After initial mild symptoms, pneumococci can asymptomatically colonize mice. Mild H1N1 infection of colonized mice did not result in a severe pneumonia but enabled lung incursion of pneumococci in 50% of cases. A recent study demonstrated that pneumococci have to adapt to the hostile environment of the IAV-infected lungs, which is characterized by hyper-inflammation and capillary leakage with efflux of antioxidants into the alveolar space^28^. Furthermore, in vivo studies linked the potential of pneumococci to cause severe co-infections to the capsular serotype^29^. Particularly invasive capsular serotypes cause severe co-infections. However, this phenomenon seems to be rather strain-specific suggesting that other pneumococcal factors are also involved^29^. Nonetheless, it was also demonstrated that non-invasive strains could cause lethal co-infections^11^. Of note, all studies mentioned above used (i) mouse-adapted IAV strains, which readily cause severe tissue damage and (ii) did not involve colonization of mice prior to viral application. Furthermore, studies have shown that severe co-infections are particularly linked to individuals with comorbidities, elderly people, pregnant women, and children under the age of one^7^. Further studies using a co-infection model equivalent to the one established in comorbid or elderly mice are warranted.

Most studies on co-infections deal mainly with one immune cell type and to the best of our knowledge; this is the first study which assesses the complexity of such infections by analyzing several phagocytic immune cells. In general only few differences in disease progression and immune cell composition of the lungs were observed between single IAV and co-infected mice. This is most likely due to (i) order of infection (first colonization followed by viral infection), (ii) the time point of the analysis (early asymptomatic vs. late severe phase of the infection), and (iii) the low virulence of the chosen viral strain. Nevertheless, our mouse model revealed significant immune responses to all types of infections, which might partially explain the non-lethal phenotype in the majority of human population. In contrast to a severe pneumococcal infection, H1N1 infection of the colonized and non-colonized mice led to a full recovery after a bi-phasic course of infection. To track potential explanations for the observed infection phenotype, we analyzed local and systemic immune responses. Irrespective of the infection type, high cytokine levels mostly associated with the myeloid immune cell compartment were observed. One of the main features of CCL2/MCP-1 is the recruitment of monocytes to the site of infection^30,31^. In line with this, an increase of total monocytes was noted in the lungs of all infected mice. IL-1 family cytokines stimulate CCL2/MCP-1 and CXCL1 expression^32,33^, and elevated levels of CXCL1 are linked to the recruitment of neutrophils^34^. IL-1β was mostly detected in the lungs of mice challenged with pneumococci and consequently, elevated neutrophil influx was found in single bacteria and co-infected animals. The effect of local IL-1β production can be further attributed to the fact that pneumococci are potent inducers of inflammasome activation and subsequent IL-1β release^35,36^. Furthermore, high IL-6 levels were observed in lungs of all infected mice. IL-6 production is mainly associated with highly pathogenic IAV viruses^37^ and has a protective role in IAV infections^38,39^. Our results show that even mild H1N1 infection is a potent inducer of protective IL-6 levels.

On the cellular level, impaired neutrophil recruitment to the lungs is associated with bacterial overgrowth in co-infections^40^. Our results show a neutrophil-independent immune response to a viral infection underlining earlier findings stating that monocytes rather than neutrophils play a crucial role in IAV infections^41^. However, IAV infections induce an apoptotic death of monocytes^42^, which is most likely reflected by a decrease of non-classical monocytes in mice lungs. As a potential compensation mechanism, we observed elevated frequencies of classical monocytes. Lung resident immune cells, including AMs, fulfill an important role in tissue homeostasis and resolution of the inflammation^9^. It has been reported that IAV infections deplete AMs^10,43,44^. Our analyses demonstrated that total AM and IM levels remained unaffected at the early onset of viral infections. This fact is further supported by in vitro analyses. Bacteria-eliminating properties of human and mouse monocytes/macrophages were not impaired in H1N1 infected cells. In contrast, reduced frequencies of both cell types were detected in single bacterial infection. IMs are most likely involved in inducing an anti-inflammatory state through IL-10 production and, thereby, inhibiting DC maturation and activation^45^. pDCs and cDCs were shown to have redundant and yet essential functions in viral infections of the lung^15^. pDCs limit viral replication and spread via type I and III interferon production^46^. However, it was demonstrated that infection with the pandemic H1N1 leads to a persistent depletion of pDCs^47^. In our experimental approach, cDC counts were reduced in single viral or pneumococcal infection, while pDC counts remained unaffected. In contrast, co-infections were characterized by elevated numbers of pDCs and unchanged cDC counts.

To initiate adaptive immune responses, it is of crucial importance for cells of the innate immune system to upregulate MHCII expression^48^. Furthermore, upregulation of CCR2 is an essential mechanism for cell trafficking from bone marrow to the site of infection^24^. Hence, upregulation of these two molecules is indicative of cell activation. We observed mainly bacteria-driven expansion of MHCII^+^ and CCR2^+^ vascular neutrophils, AMs, and CCR2^+^ IM1 populations. Furthermore, the majority of analyzed cells, except for monocytes, displayed an activated phenotype. Whether elevated CCR2 expression is beneficial or harmful is still under debate. Several studies have linked influx of CCR2^+^ cells to lung injury in mainly IAV-mediated lung infections^49–51^. In contrast, CCR2^+^ cells are important for bacterial clearance^52–54^. At this stage, we can only speculate that the observed downregulation of CCR2 on monocytes and simultaneously increased expression on other cells might benefit bacterial clearance in healthy mice resulting in a full recovery from single viral and co-infections.

In conclusion, our results demonstrate that the early onset of bacterial and viral co-infections is associated with cytokine production, an activation of resident macrophages, and an influx of mainly activated neutrophils. In contrast, infiltrating monocytes display a presumably suppressive phenotype. Such immune responses to a mild virus infection and a colonizing pneumococcal strain might contribute to the clearance of the pathogens in healthy mice. Future studies tracking the local and systemic innate immune responses as a whole and elucidating the role of comorbidities or high age are warranted.

## Methods

### Ethics statement

Buffy coats of blood provided by the blood bank at the University Medicine Greifswald were used. The buffy coats were provided anonymously. The ethical research committee at the University Medicine Greifswald approved the study (ref. no: BB 014/14). All experiments were carried out in accordance with the approved guidelines.

All animal experiments were carried out in accordance with the regulations of the German Society for Laboratory Animal Science (GV-SOLAS), the European Health Law of the Federation of Laboratory Animal Science Associations (FELASA) and in compliance with the ARRIVE guidelines. All experiments were approved by the Landesamt für Landwirtschaft, Lebensmittelsicherheit und Fischerei Mecklenburg-Vorpommern (LALLFV M-V, Rostock, Germany; permit no. 7221.3-1.1-032/17).

### Bacterial and viral strains

*Streptococcus pneumoniae* 19F (EF3030), a nasopharynx isolate from a child with frequent otitis media episodes^55,56^, was kindly provided by Anders P. Håkansson (Lund University, Sweden). 19F was grown on blood agar plates (Oxoid) and cultivated to mid-log phase (optical density [OD]_600_, 0.35–0.40) in Todd-Hewitt broth (Carl Roth) supplemented with 0.5% (w/v) yeast extract (Carl Roth) at 37 °C and 5% CO_2_. Influenza virus A/Bavaria/74/2009 (H1N1) was propagated as described by Eisfeld and colleagues^57^.

### Mice infections, monitoring, and sampling

To ensure reproducibility of the results, two independent experiments with different infectious conditions were performed. Groups of 4–12 female C57BL/6J mice (8–12 weeks old; Janvier Labs) were intranasally colonized or infected under ketamine/xylazine anesthesia with *S.* *pneumoniae* 19F or H1N1. For colonization with *S.* *pneumoniae* 19F, 20 µl PBS containing 1 × 10^7^ CFU were administered. For pneumonia, 1 × 10^8^ CFU in 20 µl PBS were applied. For viral infections, 42 µl PBS containing 10,000–250,000 PFU were used. Control mice were mock-treated with an equivalent volume of PBS. Animals were observed daily for weight and clinical score monitoring (Table S1).

At different time points, mice were euthanized with isoflurane and blood, BALF, NAL, and lungs were harvested. BALFs were obtained by rinsing the lungs with 1 ml PBS. NALs were collected by rinsing the nasopharyngeal cavity with 1 ml PBS through the trachea. Blood was collected through cardiac puncture. For differential blood count, 2.5 µl of blood were used. The blood smear was stained with a Pappenheim staining kit (Carl Roth). Plasma was obtained by centrifugation (10 min, 1000×*g*) and stored at − 80 °C.

To obtain single cell suspensions for flow cytometry, lungs were transferred into a 2 ml tube (Eppendorf), minced with sterile scissors in 1 ml digestion solution (1.5 mg ml^−1^ collagenase A, 0.25 mg ml^−1^ DNase (both Sigma Aldrich) in RPMI (HyClone)), and incubated for 1 h at 37 °C with gentle shaking (1000 rpm). To stop the digestion process and to obtain bacteria free cell suspensions, FCS and antibiotic [Penicillin G (20 μg ml^−1^) and gentamicin (120 μg ml^−1^)] were added to the samples, respectively. The resulting cell suspensions were strained through a 70 µm cell strainer and treated with RBC lysis solution.

### Flow cytometry

Prior to the cell staining, unspecific binding of immunoglobulins was blocked using 1 µg TruStain FcX PLUS (BioLegend) according to manufacturer’s instructions. All incubation steps with titrated amounts of monoclonal antibodies were carried out for 15 min at 4 °C in the dark. Washing steps were included between each staining step. Before analysis, cells were fixed with the True-Nuclear Transcription Factor Buffer Set (BioLegend) according to manufacturer’s instructions. Antibodies are summarized in Table S2. Peripheral cells were labelled via i.v. injection of 3 µg anti-CD45 antibody (30-F11, APC) 3 min prior to euthanization^58^. The gating strategy is shown in supplementary Fig. S3. Data were acquired with a LSR Fortessa flow cytometer using FACS DIVA Software and analyzed using FlowJo version 10 software (all BD Bioscience).

### Measurement of inflammation in body fluids

Cytokine concentrations of digested lung supernatants and plasma were measured via LEGENDPlex mouse inflammation panel (13-plex) kit (BioLegend) according to manufacturer's instructions. Data were acquired with a FACSAria III cell sorter using FACS DIVA Software (both BD Bioscience) and analyzed using LEGENDPlex software (BioLegend). General inflammation in BALF was determined using Bradford reagent (Sigma-Aldrich) according to manufacturer's instructions.

### Eukaryotic cells, culture conditions, and infections

To isolate human monocytes, PBMCs were isolated from buffy coats by Lymphoprep (Axis-Shields) gradient centrifugation. Cells were allowed to adhere in cell culture flasks (Corning) for 30 min at 37 °C in serum free RPMI1640 media (HyClone). The non-adherent cells were removed by washing with PBS (HyClone). The remaining monocytes were detached and 1 × 10^6^ cells/well were seeded in 6-well plates (Corning) to allow the monocytes to rest over-night in cell culture media containing 10% (v/v) FCS (Invitrogen). Expression of CD14 and CD16 by these cells was confirmed via flow cytometry.

Human monocyte-derived macrophages were generated by culturing primary human monocytes in 6-well plates for 6 days at a density of 2–3 × 10^6^ cells/well, with a media change on day 4. Monocytes were differentiated to M1 macrophages in cell culture media containing 25 ng ml^−1^ GM-CSF for 6 days, followed by LPS (100 ng ml^−1^) stimulation for additional 2 days. Monocyte-derived M2 macrophages were generated by stimulation of monocytes in cell culture media containing 50 ng ml^−1^ M-CSF for 6 days, followed by additional IL-4 (20 ng ml^−1^) stimulation for 2 days. J774A.1 mouse monocytes/macrophages (ATCC TIB-67) were cultured in RPMI1640 containing 10% (v/v) FCS. All cells were cultured under 37 °C and 5% CO_2_ atmosphere.

All infections were performed at a multiplicity of infection (MOI) 50 in a final volume of 3 ml of the respective media. Intracellular bacteria were quantified using the antibiotic protection assay^59^. For the assay, the cells were washed and infected with pneumococci. For the assessment of intracellular bacteria, 4 h after infection (t_−1_), the cells were washed with PBS and incubated with media supplemented with penicillin G (20 μg ml^−1^) and gentamicin (120 μg ml^−1^) for additional 1 (t_0_) to 5 h (t_4_) or 24 h 1 (t_23_). Subsequently, the cells were washed and lysed, and the CFU counts were determined.

### Bacterial and viral quantification in mice samples

To quantify bacterial load in mice, NAL, BALF or lung homogenates were serially diluted and plated on blood agar plates (Oxoid). The remaining cell-free BALF and NAL were stored for further analyses at − 80 °C.

To quantify viral load in mice, total RNA was isolated from the lungs using the RiboPure RNA purification Kit (Ambion) according to manufacturer's instructions. cDNA synthesis was performed using Superscript first-strand synthesis system for RT-PCR (Invitrogen). Quantitative RT-PCR amplification was performed with the SYBR GreenER Kit (BioRad). The levels of *β-actin* transcription were used for normalization. The following primers were used: NP-for, 5′-TCCGTCCTTCATTGTTCCCG-3′; NP-rev, 5′-TCCCACAAGAGGGGTCCAGA-3′; m-betaAct-for: 5′-AAATCTGGCACCACACCTTC-3′; m-betaAct-rev, 5′-GGGGTGTTGAAGGTCTCAAA-3′.

### Statistics

Statistical significance of differences between all treatment groups was determined using the Kruskal Wallis test with Dunn’s multiple comparison post-test. The presented figures depict comparisons to PBS control group. Statistics were performed using GraphPad Prism version 7. A *p* value less than 0.05 was considered significant (*p < 0.05; **p < 0.01; ***p < 0.001).

## Supplementary Information

Supplementary Information.
