# Supplementary material for: Innate immune responses at the asymptomatic stage of influenza A viral infections of Streptococcus pneumoniae colonized and non-colonized mice
Source: Sci Rep. 2021 Oct 18;11:20609. doi: 10.1038/s41598-021-00211-y (PMC8523748; doi:10.1038/s41598-021-00211-y)
Supplement: Supplementary file 1 — Supplementary Information. [file 41598_2021_211_MOESM1_ESM.pdf]

Supplementary Material

Innate immune responses at the asymptomatic stage of influenza A viral infections of *Streptococcus pneumoniae* colonized and non-colonized mice

Fabian Cuypers[1], Alexander Schäfer[2], Sebastian B. Skorka[1], Surabhi Surabhi[1], Lea A. Tölken[1], Antje D. Paulikat[1], KoInfekt Study Group, Thomas P. Kohler[1], Saskia A. Otto[3], Thomas C. Mettenleiter[2], Sven Hammerschmidt[1#], Ulrike Blohm[2], Nikolai Siemens[1#]

[1]Department of Molecular Genetics and Infection Biology, University of Greifswald, Greifswald, Germany

[2]Institute of Immunology, Friedrich-Loeffler-Institut, Federal Research Institute for Animal Health, Greifswald - Island of Riems, Germany.

[3]Institute for Marine Ecosystem and Fisheries Science (IMF), Center for Earth System Research and Sustainability (CEN), University of Hamburg, Hamburg, Germany

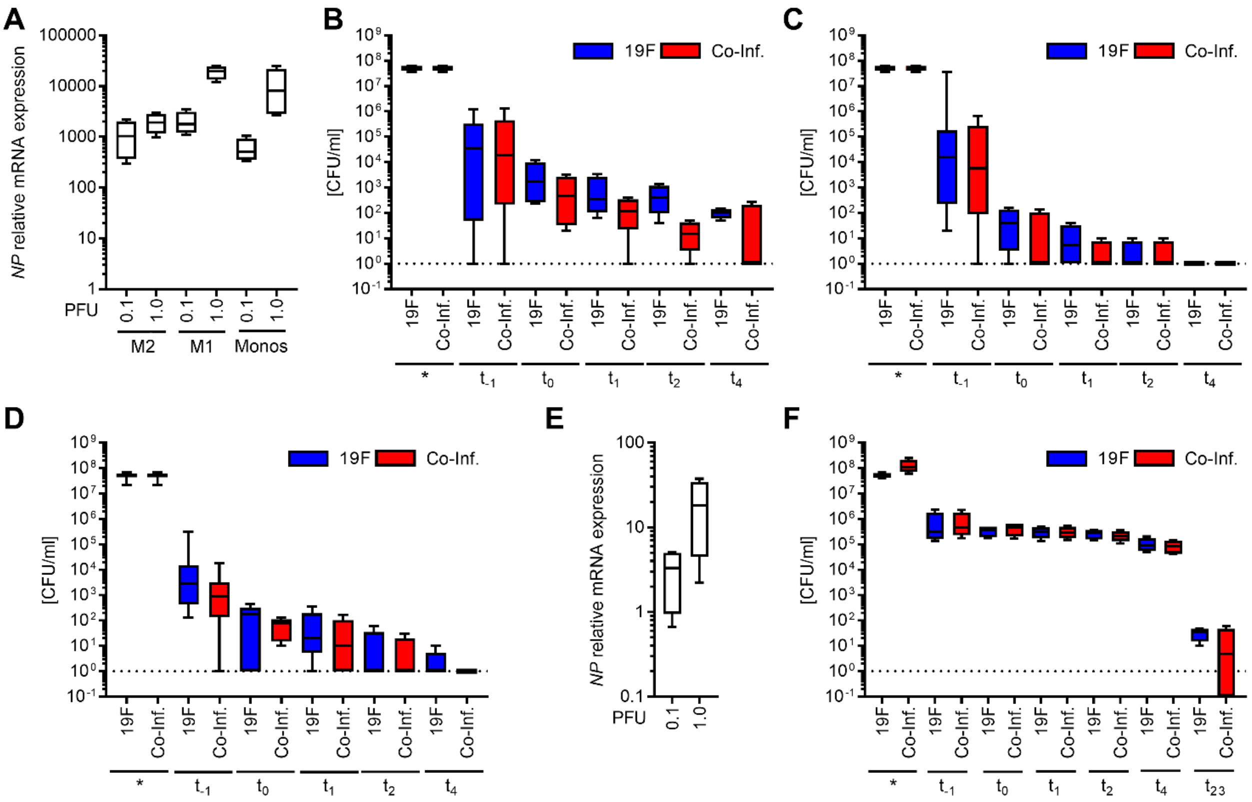

Figure S1. H1N1 infection does not impact pneumococcal clearance by professional phagocytes. (**A**) Primary human monocyte-derived macrophages of anti-inflammatory (M2) and pro-inflammatory (M1) phenotype and monocytes (Monos) were infected with indicated MOI of H1N1 and viral infection was confirmed via *NP* mRNA detection 24 h post infection. (**B**) Human primary M2 macrophages, (**C**) M1 macrophages, and (**D**) monocytes were infected with H1N1 (MOI 0.1) or left untreated for 24 h and subsequently infected with pneumococcal strain 19F (*, bacterial inoculum; t_{-1}, 4 h of infection/1 h prior to antibiotic treatment; t_0, 4 h of infection + 1 h antibiotic treatment; t_1, 4 h of infection + 2 h antibiotic treatment; t_2, 4 h of infection + 3 h antibiotic treatment; t_4, 4 h of infection + 5 h antibiotic treatment; t_{23}, 4 h of infection + 25 h antibiotic treatment). Intracellular pneumococcal CFU counts were determined at indicated time points. (**E**) Mouse J774 monocytes/macrophages were infected with indicated MOI of H1N1 and viral infection was confirmed via *NP* mRNA detection 24 h post infection. (**F**) J774 cells were infected with H1N1 (MOI 0.1) or left untreated for 24 h and subsequently infected with pneumococcal strain 19F. Intracellular CFU counts were determined at indicated time points. The data are displayed as box plots from four independent experiments (n=4).

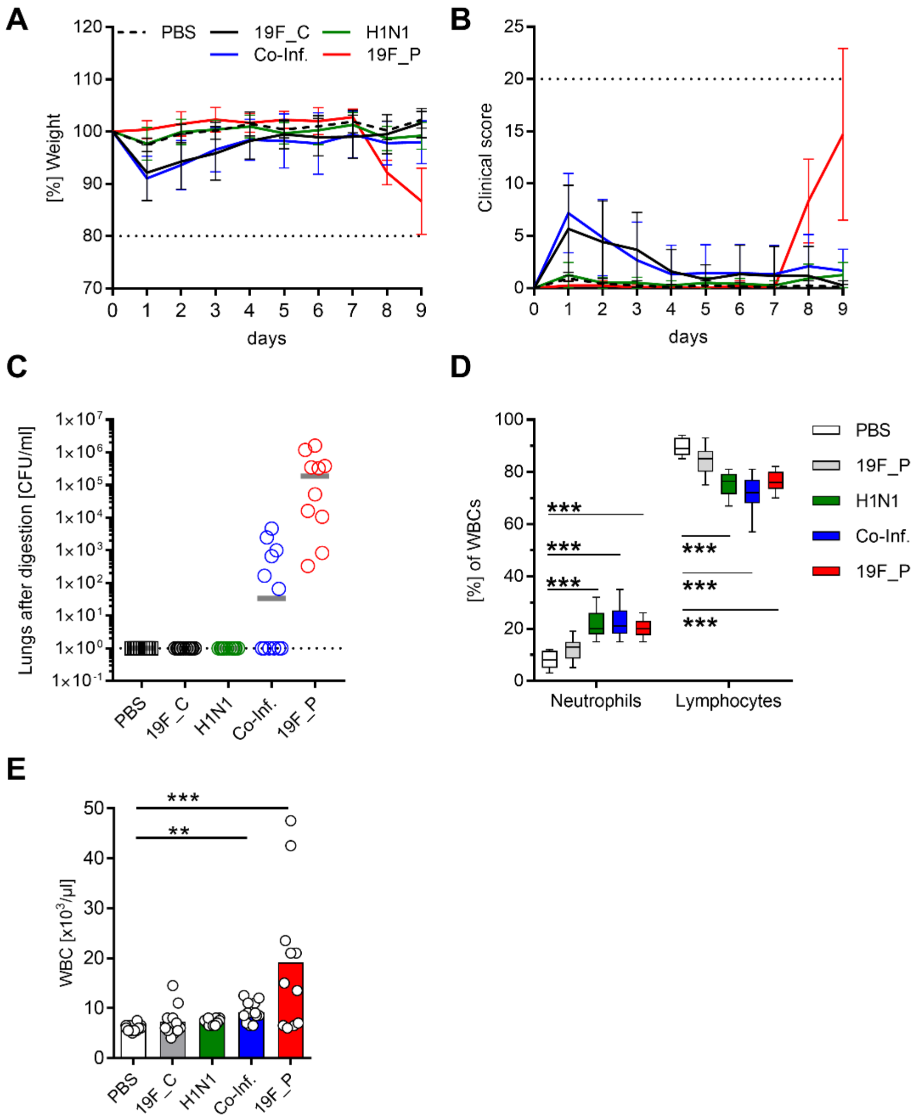

Figure S2. Single viral and co-infections of C57BL/6J mice do not cause clinical symptoms during the first two days. Female C57BL/6J mice were intranasally colonized with *S. pneumoniae* 19F (1×10^7 CFU) for seven days followed by a subsequent mild H1N1 infection (1×10^5 PFU; Co-Inf.). PBS challenged (PBS), only colonized (19F_C), and only H1N1 infected (H1N1) mice served as controls. In addition, severe pneumococcal pneumonia was induced via intranasal inoculation of 1×10^8 CFU of *S. pneumoniae* 19F (19F_P). (**A**) Weight and (**B**) clinical score were monitored over a period of nine consecutive days. (**C**) Bacterial counts obtained from digested lungs. (**D**) WBC counts obtained from the blood. (**E**) WBC counts obtained from digested lungs. Two independent experiments with six mice per group (total: n=12) were performed. Mean values ±SD are displayed (A-B).Each dot represents one mouse and horizontal lines or bars display mean values (C and E). The data in (D) are displayed as box plots. The level of significance between the groups was determined using Kruskal Wallis test with Dunn's multiple comparison post-test (*, p<0.05; **, p<0.01; ***, p<0.001).

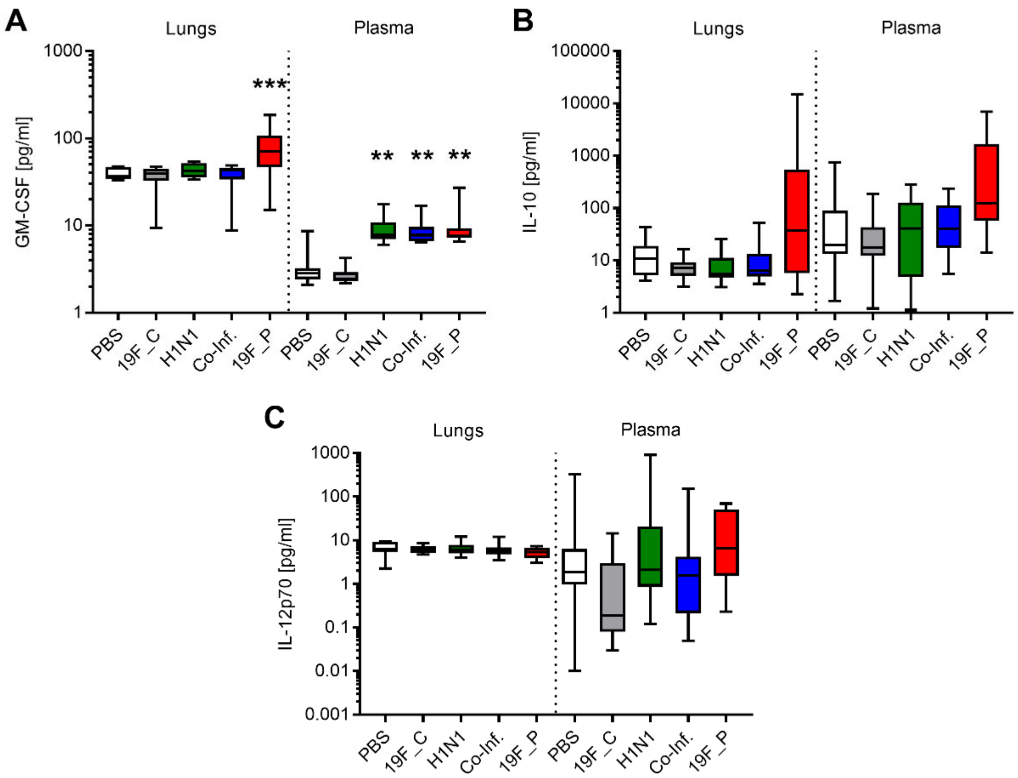

Figure S3. Local and systemic cytokine production in response to different infections. Female C57BL/6J mice were sacrificed at day nine post infections and (**A**) GM-CSF, (**B**) IL-10, and (**C**) IL-12p70 levels were determined in lungs and plasma. Two independent experiments with six mice per group (total: n=12) were performed. The data are displayed as box plots. The level of significance between the groups was determined using Kruskal Wallis test with Dunn's multiple comparison post-test (*, p<0.05; **, p<0.01; ***, p<0.001).

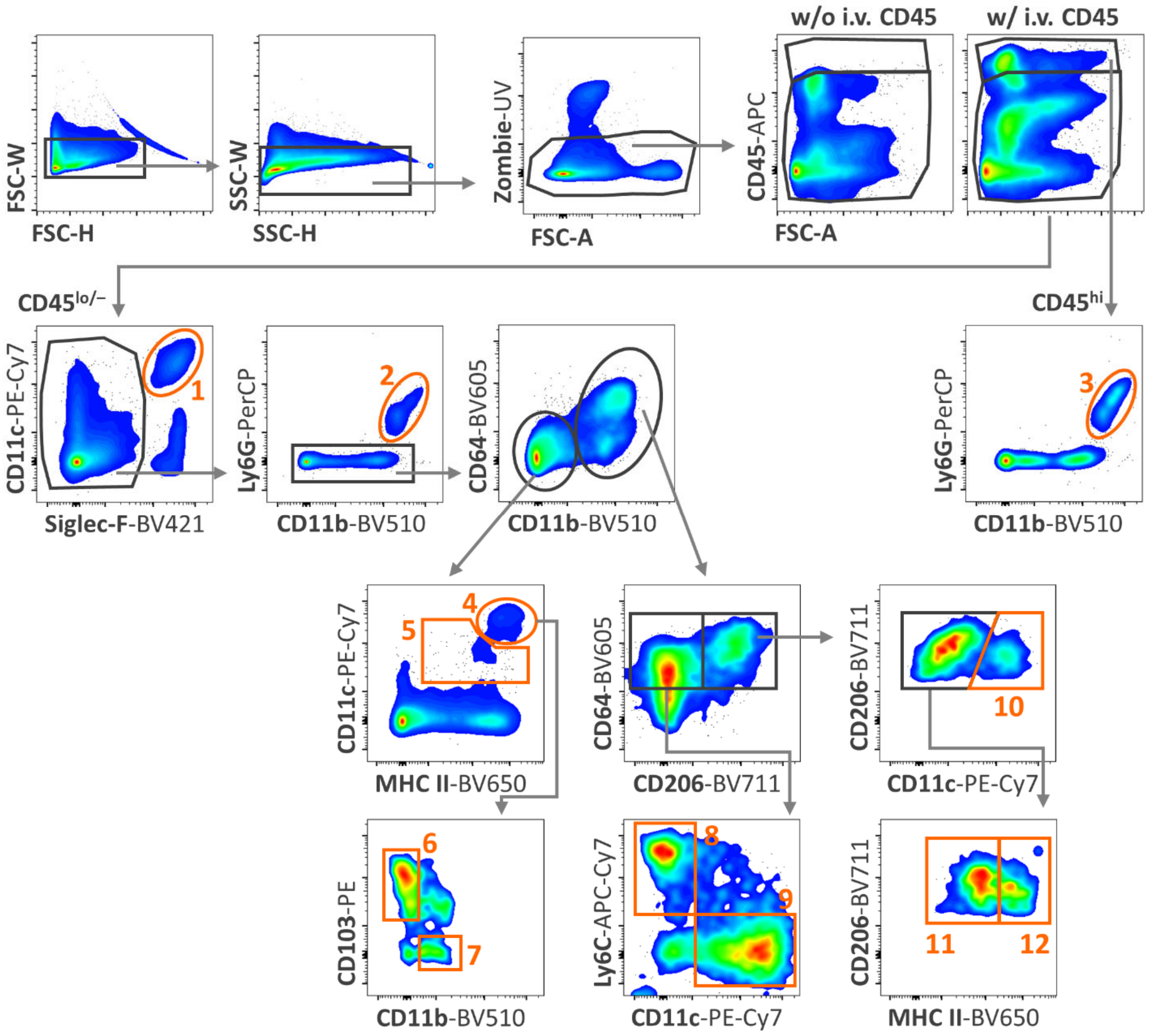

Figure S4. Gating strategy used to identify myeloid cell subsets in the vascular and alveolar/interstitial compartments of the lung. Doublets were initially excluded by consecutive FSC-W/FSC-H and SSC-W/SSC-H gating. Dead cells were excluded by using the Zombie UV Fixable Viability Kit. CD45^{hi} cells were defined as cells from the vascular lung compartment and CD45^{lo} cells were linked to the alveolar/interstitial compartment. Innate immune cell subsets were further classified as follows: (1) alveolar macrophages (AMs, CD11c^{+}Siglec-F^{+}), (2) CD45^{lo} neutrophils (CD11b^{+}Ly6G^{+}), (3) CD45^{hi} neutrophils (CD11b^{+}Ly6G^{+}), (4) conventional dendritic cells (cDCs, CD64^{-}CD11b^{-}CD11c^{+}MHCII^{+}), (5) plasmacytoid DCs (pDCs, CD64^{-}CD11b^{-}CD11c^{int}MHCII^{+}), (6) CD103^{+}CD11b^{-}cDCs, (7) CD11^{+}CD103^{-}cDCs, (8) inflammatory monocytes (CD11b^{hi}CD64^{int}CD206^{-} Ly6C^{+}CD11c^{-}), non-classical monocytes (CD11b^{hi}CD64^{int}CD206^{-} Ly6C^{-}CD11c^{+}), and interstitial macrophages (CD11b^{hi}CD64^{int}CD206$^{int/+}$; (12) CD11c^{-}CD206^{+}MHCII^{int} (IM1), (11) CD11c^{-}CD206^{+}MHCII^{+} (IM2), and (10) CD11c^{+}CD206^{int} (IM3)).

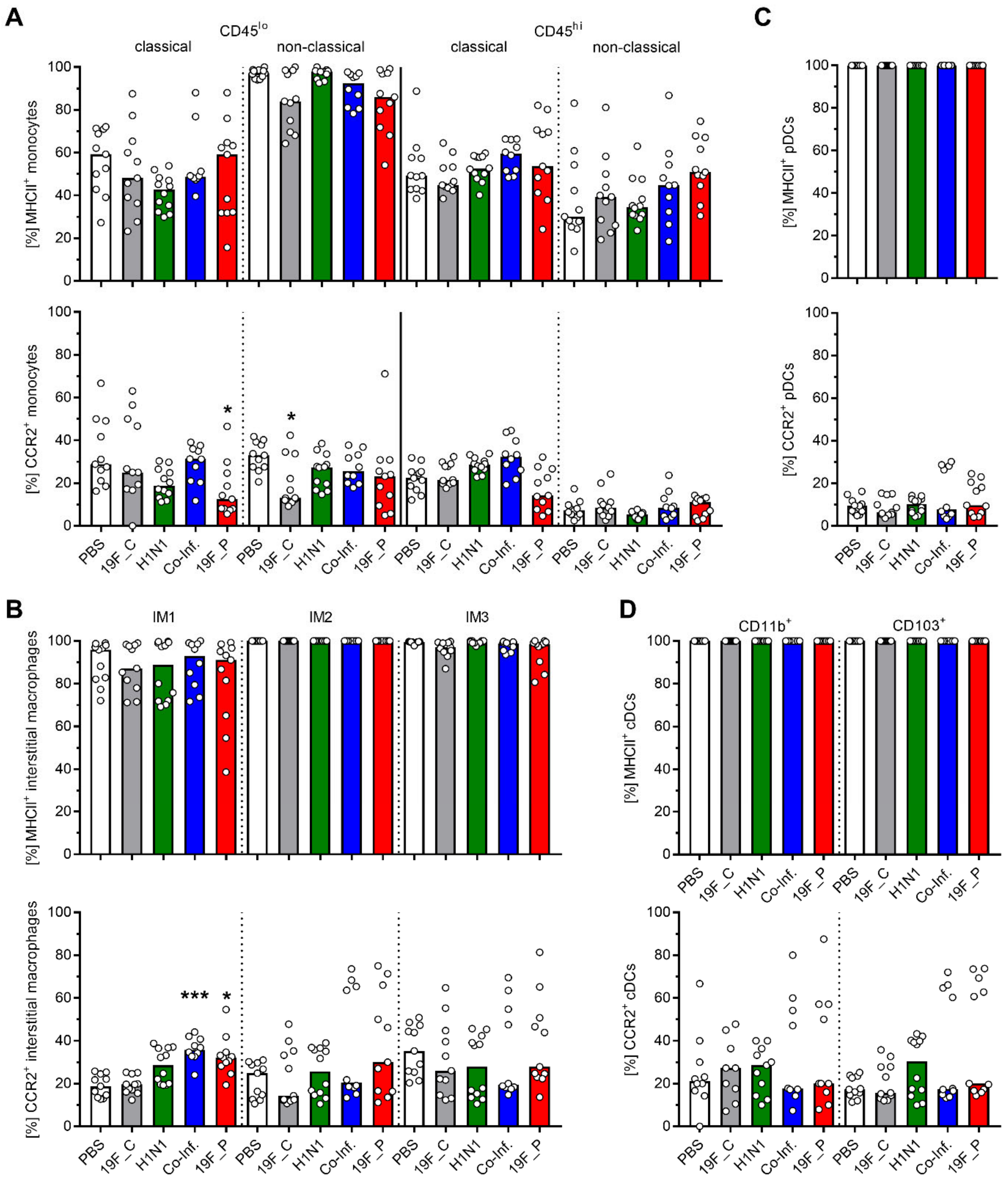

Figure S5. Frequencies of MHCII+ and CCR2+ innate immune cells in response to bacterial and viral infections. The infections were performed as displayed in Figure 5A. The data display expression of MHCII (upper panels) and CCR2 (lower panels) on (**A**) monocytes, (**B**) interstitial macrophages (IMs), (**C**) plasmacytoid dendritic cells (pDCs), and (**D**) conventional dendritic cells (cDCs). Each dot represents one mouse (n≥10). The level of significance between the groups was determined using Kruskal Wallis test with Dunn's multiple comparison post-test (*, *p*<0.05; **, *p*<0.01; ***, *p*<0.001).

Table S1: Scoring used for clinical monitoring. Animals were observed daily. A total score of 20 resulted in termination of the experiment.

**Weight loss**	**Points**
<5%	1 pt
5-10%	5 pt
11-20%	10 pt
>20%	20 pt
**general conditions**	
smooth fur and shiny, body openings clean, eyes clear	0 pt
fur defects (body maintenance)	1 pt
blunt fur, ruffled, dirty body openings, turbid eyes, slightly higher tonicity	5 pt
dirty fur, sticky or wet body openings, unnatural stance, turbid eyes, high tonicity	10 pt
cramps, palsy, respiratory sounds, animal is cold	20 pt
**spontaneous reactions**	
normal behavior (sleeping, reaction towards blowing and touching, social contacts, nosiness)	0 pt
minor aberration from normal behavior	1 pt
unnatural behavior, limited motor function, hyper kinetics	5 pt
self-isolation, lethargy, pronounced hyper kinetics, coordination disorder	10 pt
pain sounds when being touched, self-amputation (auto-aggression, autonomy)	20 pt

Table S2: Antibodies used for flow cytometry analyses.

Product code	Antibody	Host species	Clone	Company
103112	APC anti-mouse CD45	Rat	30-F11	BioLegend
128026	APC/Cyanine7 anti-mouse Ly6C	Rat	HK1.4	BioLegend
396414	Brilliant Violet 421TM anti-human/mouse Granzyme B Recombinant	Mouse	QA18A28	BioLegend
155509	Brilliant Violet 421TM anti-mouse CD170 (Siglec F)	Rat	S17007L	BioLegend
101263	Brilliant Violet 510TM anti-mouse/human CD11b	Rat	M1/70	BioLegend
139323	Brilliant Violet 605TM anti-mouse CD64 (FcγRI)	Mouse	X54-5/7.1	BioLegend
107639	Brilliant Violet 605TM anti-mouse I-A/I-E	Rat	M5/114.15.2	BioLegend
118129	Brilliant Violet 605TM anti-mouse TCR γ/δ	Armenian Hamster	GL3	BioLegend
141727	Brilliant Violet 711TM anti-mouse CD206 (MMR)	Rat	C068C2	BioLegend
135231	Brilliant Violet 711TM anti-mouse CD279 (PD-1)	Rat	29F.1A12	BioLegend
150621	Brilliant Violet 785TM anti-mouse CD192 (CCR2)	Rat	SA203G11	BioLegend
121406	PE anti-mouse CD103	Armenian Hamster	2E7	BioLegend
117318	PE/Cy7 anti-mouse CD11c	Armenian Hamster	N418	BioLegend
104512	PE/Cy7 anti-mouse CD69	Armenian Hamster	H1.2F3	BioLegend
127654	PerCP anti-mouse Ly6G	Rat	1A8	BioLegend
652424	PerCP/Cyanine5.5 anti-mouse Ki-67	Rat	16A8	BioLegend
156604	TruStainFcXTM PLUS (anti-mouse CD16/32)	Rat	S17011E	BioLegend
423108	Zombie UVTM Fixable Viability Kit			BioLegend
563565	BUV395 anti-mouse CD3e	Armenian Hamster	145-2C11	BD Biosciences
